# Sex dimorphism controls dysbindin-related cognitive dysfunctions in mice and humans with the contribution of COMT

**DOI:** 10.1038/s41380-024-02527-3

**Published:** 2024-03-26

**Authors:** Federica Geraci, Roberta Passiatore, Nora Penzel, Samuele Laudani, Alessandro Bertolino, Giuseppe Blasi, Adriana C. E. Graziano, Gianluca C. Kikidis, Ciro Mazza, Madhur Parihar, Antonio Rampino, Leonardo Sportelli, Nicolò Trevisan, Filippo Drago, Francesco Papaleo, Fabio Sambataro, Giulio Pergola, Gian Marco Leggio

**Affiliations:** 1 Department of Biomedical and Biotechnological Sciences, University of Catania, 95123 Catania, Italy; 2 Department of Translational Biomedicine and Neuroscience, University of Bari Aldo Moro, 70124 Bari, Italy; 3 Lieber Institute for Brain Development, Johns Hopkins Medical Campus, 21205 Baltimore, MD USA; 4 Psychiatric Unit - University Hospital, 70124 Bari, Italy; 5 Department of Human Genetics, Radboud University Nijmegen, 6525 GD Nijmegen, The Netherlands; 6 Department of Neuroscience (DNS), University of Padova, 35121 Padova, Italy; 7 Genetics of Cognition Laboratory, Neuroscience area, Istituto Italiano di Tecnologia, Genova, Italy; 8 Department of Psychiatry and Behavioral Sciences, Johns Hopkins University School of Medicine, 21205 Baltimore, MD USA

**Keywords:** Schizophrenia, Neuroscience

## Abstract

Cognitive dysfunctions are core-enduring symptoms of schizophrenia, with important sex-related differences. Genetic variants of the *DTBPN1* gene associated with reduced dysbindin-1 protein (Dys) expression negatively impact cognitive functions in schizophrenia through a functional epistatic interaction with Catechol-O-methyltransferase (COMT). Dys is involved in the trafficking of dopaminergic receptors, crucial for prefrontal cortex (PFC) signaling regulation. Moreover, dopamine signaling is modulated by estrogens via inhibition of COMT expression. We hypothesized a sex dimorphism in Dys-related cognitive functions dependent on COMT and estrogen levels. Our multidisciplinary approach combined behavioral-molecular findings on genetically modified mice, human *postmortem* Dys expression data, and in vivo fMRI during a working memory task performance. We found cognitive impairments in male mice related to genetic variants characterized by reduced Dys protein expression (p_Bonferroni_ = 0.0001), as well as in male humans through a COMT/Dys functional epistatic interaction involving PFC brain activity during working memory (t(23) = −3.21; p_FDR_ = 0.004). Dorsolateral PFC activity was associated with lower working memory performance in males only (*p* = 0.04). Also, male humans showed decreased Dys expression in dorsolateral PFC during adulthood (p_FDR_ = 0.05). Female Dys mice showed preserved cognitive performances with deficits only with a lack of estrogen tested in an ovariectomy model (p_Bonferroni_ = 0.0001), suggesting that genetic variants reducing Dys protein expression could probably become functional in females when the protective effect of estrogens is attenuated, i.e., during menopause. Overall, our results show the differential impact of functional variants of the *DTBPN1* gene interacting with COMT on cognitive functions across sexes in mice and humans, underlying the importance of considering sex as a target for patient stratification and precision medicine in schizophrenia.

## Introduction

The incidence, age of onset, symptoms, and response to treatment in psychiatric disorders can vary significantly between sexes [1]. However, many clinical and preclinical investigations do not include sex as a biological component in the experimental design. For this reason, the US - National Institute of Health published a policy in 2015 encouraging the inclusion of females in both clinical and basic research, with the ultimate goal of harmonizing results of human and preclinical research and, ultimately, providing new insights on the etiology and treatment of mental disorders in an era of more sex-tailored medicine [2]. Of relevance to the study of psychiatric disorders, sex differences in relationship with clinical symptoms, course, and treatment outcome have been consistently reported in schizophrenia [3]. Male patients show an earlier age of onset, a higher propensity to negative symptoms, lower social functioning, and more frequent co-morbid substance abuse than females. Female patients show a relatively later onset with more affective symptoms. Therefore, it has been hypothesized that estrogens may protect against schizophrenia throughout life [4, 5]. Cognitive dysfunctions are core-enduring symptoms in schizophrenia that contribute dramatically to poor functional outcomes in patients and constitute an unmet therapeutic need [6]. Also, for cognitive symptoms of schizophrenia, a greater cognitive impairment spanning several cognitive domains, including working memory, has been reported in male relative to female patients [7]. Inter-individual differences in cognitive abilities in normal individuals and patients with schizophrenia have been associated with variations in the *DTNBP1* gene, encoding dysbindin-1 protein (Dys), which is involved in schizophrenia neuropathology and cognitive function during treatment with antipsychotic drugs [8–10]. More specifically, Dys plays a role in the regulation of brain development [11, 12] influencing synapse formation and maintenance, dopamine D2-like intracellular receptor trafficking [9, 13–15] and neurotransmitter release [14, 16–18]. Dys functions differ between sexes [19]. Reduced expression levels of Dys mRNA and protein in brain areas relevant to schizophrenia, including the prefrontal cortex (PFC), the hippocampus, and the nucleus accumbens, have been reported in patients [14, 20]. In PFC, Dys contributes to the pattern of excitability in PFC microcircuits [21] through a functional epistatic effect with Catechol-O-methyltransferase (COMT) [22], an enzyme that is involved in metabolizing various catecholamine neurotransmitters, including dopamine. Lower COMT activity combined with lower *DTNBP1* expression is associated with poorer performance in mice and lower PFC efficiency measured with functional magnetic resonance imaging (fMRI) during the performance of a working memory task in humans, compared with carriers of a single genetic variation either in *COMT* or *DTNBP1* genes. These mechanisms have been associated with the genetic risk for schizophrenia, particularly, with impaired working memory [15, 23], and altered cognitive responses to antipsychotic drugs [9, 15, 24]. Estrogens have been proposed as mediating factors of the relationship between sex and cognition and antipsychotic treatment response via dopamine signaling modulation [25, 26]. Estrogens can control COMT activity [27, 28] through estrogen response elements (ERE) sequences that are present in the promoter of the *COMT* gene [29] and inhibit its transcription [30, 31]. Indeed, estrogen deprivation can result in increased COMT activity and decreased dopaminergic functioning, lowering the sensitivity of dopamine D2 receptors and, as a result, lowering the efficacy of (D2-receptor binding) antipsychotics [32, 33]. Moreover, ERR1 (estrogen-related receptor 1; coded by the *ESRRA* gene) has been associated with the modulation of the expression of genes co-expressed with *DRD2* in the human dorsolateral PFC (DLPFC) [34]. Genetic variants within this co-expressed gene set are associated with neuroimaging, behavioral and clinical phenotypes associated with schizophrenia [35]. Although no direct interaction between estrogens and Dysbindin has been shown from a molecular point of view, the effects of estrogen on dopamine signaling [36] and the role of COMT and Dys and their functional epistatic interaction in the modulation of prefrontal cognitive functions in both humans and mice [22] are strongly suggestive of a pathway of convergence of COMT, Dys, and estrogens that can mediate sex differences in cognition. Analyzing the combined effect of the sex dimorphism of Dys on prefrontal working memory through the interaction with COMT during estrogenic periods during lifespan may reveal schizophrenia-related pathophysiological processes trackable across age stages and sexes.

To test this hypothesis, we evaluated sex-driven differences in behavioral phenotypes in Dys-related cognitive domains using a murine genetic model carrying Dys hemideletion (Dys + /+; Dys + /− male and female mice). To understand possible mechanisms of sexual dimorphism in dysbindin-related cognitive dysfunctions, we investigated the role of COMT in Dys mice since its activity depends on estrogen levels [25, 26]. We tested the functional epistatic interaction between the *DTNBP1* haplotype (Dys Hap) previously associated with reduced Dys mRNA expression in the human brain [22], i.e., the three-marker rs2619538-rs3213207-rs1047631, and the *COMT* common variant *rs4680* that has been associated with working memory efficiency [22]. In particular, we evaluated the COMT immunoblotting levels in the medial PFC (mPFC) of Dys mice. Based on mice results, we assessed sex differences in Dys gene expression throughout life in humans using the analysis of human RNA sequencing data from the Lieber Institute for Brain Development (LIBD) Brain Repository to test differential mRNA expression across age stages and sexes in several brain regions. Lastly, as the DLPFC is a brain region with similar cognitive functions and connections to murine mPFC [37], we analyzed the effects of Dys Hap/COMT on the activity of DLPFC during the performance of a working memory task in humans using fMRI, hypothesizing a matching sex dimorphism in brain cognitive functioning between mice and humans.

## Methods

### Mice models

#### Mice

Experiments were carried out in 3 to 6-month-old female and male Dys heterozygous mutant mice [38, 39] (Dys + /− female *n* = 75, male *n* = 28) and their wild-type littermates (Dys + /+ female *n* = 80, male *n* = 35). Mice were housed in 2–4 per cage in a room with controlled temperature (21 ± 1 °C) and humidity (55 ± 10%) with a 12 h light/dark cycle. Food and water were available ad libitum. For details, see the Supplementary Information, Section 1.

#### Estrous cycle phases identification

Stages of the estrus cycle were identified by cytological analysis of vaginal secretion, as previously described [40–42]. For details, see the Supplementary Information, Section 1.2.

#### Surgery

Female Dys + /− and Dys + /+ mice were ovariectomized under isoflurane anesthesia, as previously described [43, 44]. The ovaries were removed through a bilateral incision at the flank level. Control Dys + /− and Dys + /+ female mice were anesthetized and subjected to sham surgery, consisting of a bilateral incision without removing ovaries. The animals were housed individually for 24 h to fully recover from surgery. One week after surgery, vaginal cytological samples were taken to verify the loss of ovarian function. Mice were randomly assigned to ovariectomy or Sham surgery.

#### Drugs

Chronic peroral administration of 17ß-estradiol (E2) administration (Sigma-Aldrich, US) was carried out as described elsewhere [45, 46]. For details, see the Supplementary Information, Section 1.1.

#### Behavioral test: temporal order recognition (TOR) Test

The TOR test was performed as previously described in [15, 47]. For details, see the Supplementary Information, Section 1.3.

#### Protein extraction and western blot analysis

Western blot analysis was carried out on proteins extracted from isolated mPFC brain area. For details, see the Supplementary Information Section 1.4.

#### Statistical analysis

Statistical analyses in mice were performed using Prism 9 (GraphPad Software, Version 9.1.1, La Jolla, CA, USA). For details, see the Supplementary Information Section 1.6.

### Humans

#### Brain postmortem study

Brain tissue data were collected at the LIBD [48, 49]. All individuals had minimal age-associated neuropathology, no substance use, and no psychiatric or neurological disorders, determined by postmortem histopathological examination, toxicology, and clinical histories, respectively. All individuals were of European or African American ancestry. We selected individual samples with the RNA Integrity Number ≥6, i.e., the optimal range for gene expression data [50]. *DTNBP1* is expressed in the DLPFC, hippocampus, caudate, putamen, nucleus accumbens, amygdala, thalamus, and midbrain of the adult human brain [51]. Thus, we first quantified the gene expression from *postmortem* samples extracted from the DLPFC, the hippocampus, and the caudate nucleus of both males and females available in the LIBD repository. Our investigation of sex differences in Dys gene expression included 261 (33% female), 276 (33% female), and 259 (31% female) samples for the analysis of the DLPFC, hippocampus, and caudate, respectively (Table 1) [48].

**Table 1:** Demographical data for each sample divided for age groups and brain regions.

Sample size – mean age in years ± SD (age range min:max) - %female			
	DLPFC	Hippocampus	Caudate
Perinatal	49–0.5 ± 1.5 (−0.5:5.3) –47%	42–0.02 ± 1.2 (−0.5:4.7) –52%	20–1.0 ± 1.4 (0:4.2) –35%
Juvenile	45–18.5 ± 13.5 (6.3:24.8) –27%	47–18.6 ± 3.6 (8.2:24.8) –23%	29–19.8 ± 3.6 (13:24.8) –35%
Younger adults	88–40.4 ± 7.2 (25.8:49.7) –30%	104–40.0 ± 7.4 (25:49.7) –31%	103–40.5 ± 7.3 (25:50) –29%
Older adults	79–59.0 ± 7.6 (50.3-84.2) –29%	83–58.9 ± 8.2 (50.1:84.2) –27%	107–61.8 ± 10.1 (50.3:89.9) –28%

*DLPFC* Dorsolateral prefrontal cortex, *SD* Standard deviation.

We derived DLPFC samples from Brodmann Area 46; the hippocampus samples from the hippocampus proper, including the dentate gyrus, CA3, CA2, CA1, and the subicular complex [50, 52]. As regards the caudate nucleus, we consider the anterior ‘head’ portion, the caudate portion most closely connected to the prefrontal cortex. For all tissues, RNA sequencing was performed via the Illumina Ribozero Kit. Age differences across brain regions were assessed using the Chi-square independence test (α < 0.05). Regional co-expression quantification and genotyping follow standard procedures [48] and are described in Supplementary Information, Section 2.1. We tested sex differences in *DTNBP1* expression in the hippocampus, DLPFC, and caudate through two-sample t tests applying the False discovery rate (FDR) correction to control for multiple comparisons (*k* = 3 regions of interest). We stratified the analysis into four age groups, i.e., perinatal (1–6 years), juvenile (12–25 years), young adults (25.1-50 years), and older adults (50.1–90 years) to account for regional specificity of *DTNBP1* expression across age groups. while interaction between sex and the COMT rs4680 genotype on DTNBP1 expression using 2way-ANOVAs. Statistical analyses were performed through R (https://www.r-project.org), version 4.1.2.

#### Brain imaging study

Two hundred and seven healthy adults of non-related European ancestry participated in an fMRI study at the University of Bari Aldo Moro (Male: *N* = 105; mean age in years±standard deviation=27.1 ± 6.9; Female: N = 102; mean age in years ± standard deviation=25.3 ± 5.8). For each participant, we assessed intelligence quotient (IQ) using the Wechsler Adult Intelligence Scale — revised [53] (Male: mean IQ scores±standard deviation=112.5 ± 11.7; Female: mean IQ scores±standard deviation=105.2 ± 10.5). Age and IQ differences between the sex groups were tested using the two-sample *t*-test (α < 0.05). The experimental protocol was approved by the local ethics committee. Written informed consent was obtained after a full understanding of the protocol according to the Declaration of Helsinki. Inclusion/exclusion criteria are described in the Supplementary Information, Section 2.2.

#### Genotype determination

Participants underwent blood withdrawal for subsequent DNA extraction from peripheral blood mononuclear cells. Details on genotype extraction and processing are reported in Supplementary Information, Section 2.3. To test the Dys Hap/COMT interaction on fMRI, we selected the single nucleotide polymorphisms in COMT and the Dys Hap based on previous literature [54]. The *COMT Val158Met, rs4680*, was not available in our sample; therefore, we selected a single nucleotide polymorphism in linkage disequilibrium named *rs4633* (D’ = 0.996, R^2^ = 0.988), a synonymous variant at codon 62 of the COMT gene. We will refer hereinafter to the carriers of the Met allele as COMT^MetCar^ compared to COMT^Val/Val^ individuals. COMT^Met/Met^ and COMT^Val/Met^ were grouped because of the small sample size of each group. Regarding the three-marker Dys Hap (*rs2619538-rs3213207-rs1047631*) at the *DTNBP1* gene locus previously associated with a pattern of cognitive-related DLPFC functional activity consistent with reduced expression [9, 22], the single nucleotide polymorphism in linkage disequilibrium *rs9296989* replaced *rs2619538* (D’ = 0.96, R^2^ = 0.84). We will refer hereinafter to the carriers of Dys Hap (G-T-T) as Dys Hap + /− in comparison to Dys Hap + /+ individuals. Dys Hap + /− and Dys Hap − /− were grouped due to the small sample size of each group.

#### The N-back task

All participants underwent an fMRI experiment completing a blocked paradigm of a previously published working memory task, the N-back task [55, 56]. Further details about the neuropsychological paradigm are reported in Supplementary Information, Section 2.4. To determine behavioral performance, we computed an efficiency rate as the ratio between the hit rate and reaction time [57]. Higher values of this index indicated better behavioral performance [58]. Behavioral differences across sexes in terms of hit rate, reaction time, and efficiency between sex groups were tested through two-sample *t*-tests (α < 0.05).

#### MRI data acquisition and processing

fMRI scans along with a structural T1-weighted scan were acquired with a General Electric (Milwaukee, WI) 3.0 Tesla whole-body scanner using a standard quadrature head coil and processed with the Computational Anatomy Toolbox (CAT12, http://dbm.neuro.uni-jena.de/cat/) and the Statistical Parametric Mapping version 12 (SPM12, http://www.fil.ion.ucl.ac.uk/spm) implemented in MATLAB R2017a (https://it.mathworks.com/). Acquisition parameters, individual-level data processing, and quality check procedures are provided in Supplementary Information, Section 2.5.

To detect significant brain activity at the group-level, we performed a three-way voxel-wise ANOVA in SPM12 on individual-level activity maps during the N-back task. The model included main effects along with two-way and three-way interactions between sex, COMT genotype, and Dys Hap. We report results masked by N-back task activity within the grey matter and thresholded at whole-brain level using the threshold-free cluster enhancement [6] (TFCE). Statistics were adjusted for multiple comparisons as the number of voxels based on the Family-Wise error rate (p_TFCE-FWE_ < 0.05). Additional control analyses on the impact of processing methods on sex-related brain activity are detailed in Supplementary Information, Section 2.6.

The individual brain activity estimates were then extracted from the significant clusters using Marsbar (http://marsbar.sourceforge.net/) and entered into *post hoc* two-sample t-tests comparing DLPFC activity on all combinations of *COMT* genotypes and Dys Hap within the sex groups (α < 0.05). To assess brain-behavior relationships, we conducted linear regressions between individual DLPFC activity estimates and working memory performance across sex groups. We used the hit rate, reaction time, and the efficiency rate as continuous predictors in three separate analyses (α < 0.05) including the *COMT* rs4633 genotype, and Dys Hap as categorical factors. Statistical analyses were performed through R, version 4.1.2.

## Results

### Dysbindin affects the cognitive functions of mice in a sex-dependent manner

**Fig. 1:**
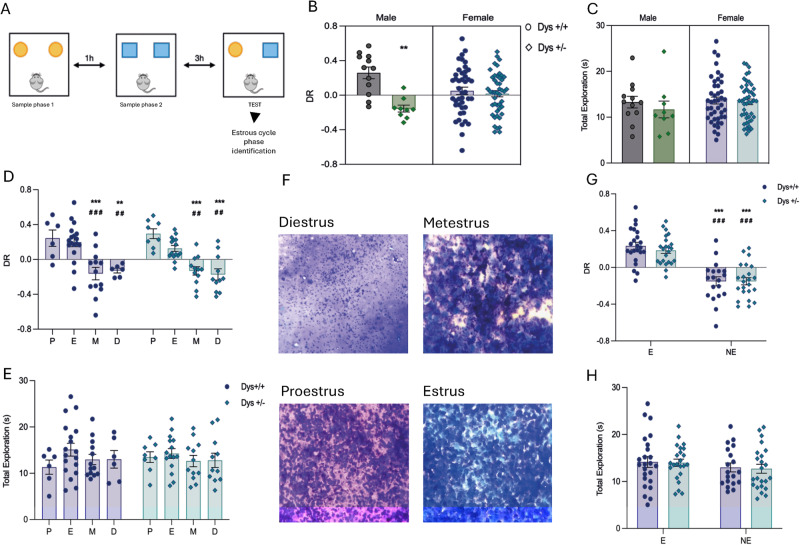
Dysbindin affects the cognitive functions of mice tested in the TOR test in a sex-dependent manner. **A** Cartoon depicting the behavioral task Temporal Order recognition. **B** Discrimination Ratio (DR). 2-Way ANOVA Gender X Genotype Interaction F(1,106) = 9.58, **p = 0.0025, Genotype effect F(1,106) = 13.20 ***p = 0.000, Gender effect F(1,106) = 0.082; *p* = 0.7746; Bonferroni’s multiple comparison test; *p* > 0.999 vs. Dys + /− female, **p = 0.0018. vs. Dys + /− male. Dys + /+ female *n* = 32; male: *n* = 12, Dys + /− female *n* = 35 male *n* = 9. **C** Total Exploration in seconds, Dys + /+ female *n* = 32; male: *n* = 12, Dys + /− female *n* = 35 male *n* = 9. 2-Way ANOVA did not reveal any difference between genotype (F(1,106) = 0.688) or gender (F(1, 106) = 0.7906. **D** Discrimination ratio (DR) analyzed according to the four phases of mice estrous cycle: Proestrus (P), Estrus (E), Metestrus (M) and Diestrus (D). Two-Way ANOVA revealed an estrous phase effect F (3,81) = 23.22, *p* < 0.0001 but not a genotype effect F(1,81 = 0.03564, *p* = 0.8507 not interaction F(3,81) = 0.5134, *p* = 0.6742. Bonferroni’s Multiple Comparison Test: **p < 0.01, ***p < 0.001. (Dys + /+: P *n* = 6, E *n* = 18, M *n* = 13, D *n* = 6; Dys + /−: P *n* = 8, E *n* = 15, M *n* = 12, D *n* = 11). **E** Total exploration in seconds, Two-way ANOVA: Estrous cycle phase F(3,81) = 1.252, *p* = 0.2965; Genotype F(1, 81 = 0.03240, *p* = 0.8576; Estrous cycle phase X Genotype F(3,81) = 0.3488, *p* = 0.7901; Dys + /+: P *n* = 6, E *n* = 18,M *n* = 13, D *n* = 6; Dys + /−: P *n* = 8, E *n* = 15, M *n* = 12, D *n* = 11). **F** Photos depicting the four phases of mice estrous cycle assessed immediately after the TOR test. **G** DR analyzed as “estrus” (E) and “non-estrus” (NE) phases. 2Way-ANOVA: estrous.cycle effect F(1,85) = 69.9 ***p < 0.0001, Genotype effect F(1,85) = 0.0822 *p* = 0.7749, Interaction E.phaseXGenotype F(1,85) = 0.0937 *p* = 0.7602. Bonferroni’s multiple comparison tests: ***< 0.0001 Dys + /+ E vs.NE, ***p < 0.0001 Dys + /− E vs. NE. **H** Total exploration time. No effect of genotype or estrous cycle phase affected the total exploration time. 2 Way-ANOVA estrous cycle effect F(1,85) = 1,571 *p* = 0.2135, Genotype effect F(1,85) = 0.051 *p* = 0.822, Interaction F(1,85) = 0.0036 *p* = 0.9525.

To selectively address the Sex × Dys interaction, we used both female and male mice with the hypofunction of the Dys gene (Dys + /− mice). This approach avoided possible confounders related to human studies, such as genetic heterogeneity, environmental effects, and pathological state. We tested both Dys + /− female and Dys + /− male mice on the TOR test which is sensitive to dopaminergic signaling within the mPFC [15, 59]. Remarkably and in contrast with results obtained in Dys + /− male mice, the Dys + /− female mice tested in the TOR test did not show differences compared to their Dys + /+ littermates (Fig. 1B; 2way-ANOVA Sex-Genotype interaction F(1,106) = 9.58, **p = 0.0025, Sex effect F(1,106) = 13.20 ***p < 0.001, Sex effect F(1,106) = 0.082; *p* = 0.7746; Bonferroni’s multiple comparison test; *p* > 0.999 vs. Dys + /− female, **p = 0.0018. vs. Dys + /− male; Dys + /+ female *n* = 32; male: *n* = 12, Dys + /− female *n* = 35 male *n* = 9), suggesting that the Dys genotype affects cognitive functions differently in a sex-biased manner. No differences in total exploration time were observed between the groups (Fig. 1C). Subsequently, to avoid any confounding factor related to the ovarian cycle and to assess the role of hormonal fluctuation throughout the estrous cycle in Dys-related cognitive performance, we tested female Dys + /+ and Dys + /− female mice in the TOR test also according to the four phases of the ovarian cycle (Fig. 1F). Indeed, cognition in women and female mice have been reported to be dependent on the estrus state [60–64]. There was a main effect of the estrous cycle with greater performance in mice that during the test were in their proestrus or estrous phase, in which E2 levels peak and begin to decline, compared to those in metestrus or diestrus, both characterized by low E2 levels (Fig. 1D; 2way-ANOVA, Bonferroni’s Multiple Comparison Test: **p < 0.01; ***p < 0.001). To further elucidate the relationship between E2 levels during the ovarian cycle and the cognitive performance of Dys mice, we separated the ovarian cycle into two macro-phases defined as the ‘estrus’ (E) period, the period that encloses proestrus (marked by a rise in estrogen) and estrus, (when estrogen levels begin to decline), and the phase defined as the ‘non-estrus’ (NE) period (metestrus and diestrus phases) [65]. This analysis revealed a main effect of the E period in the TOR test (Fig. 1G; Dys + /+ *n* = 43 (E *n* = 24; NE *n* = 19); Dys + /− *n* = 46 (E *n* = 23, NE *n* = 23); 2Way-ANOVA, Estrus cycle F(1,85) = 69.90; ***p < 0.0001; Bonferroni’s Multiple Comparison Test ***p < 0.0001). No effect on total exploration time was detected (Fig. 1E–H).

Together, these results show that Dys + /− female mice, in contrast with their male counterpart, do not show TOR memory impairment. Further, they confirm the prominent role of the interaction between E2 levels Dys genotype in modulating cognitive abilities in females.

### Ovariectomy produces cognitive impairments in female Dys + /− mice

**Fig. 2:**
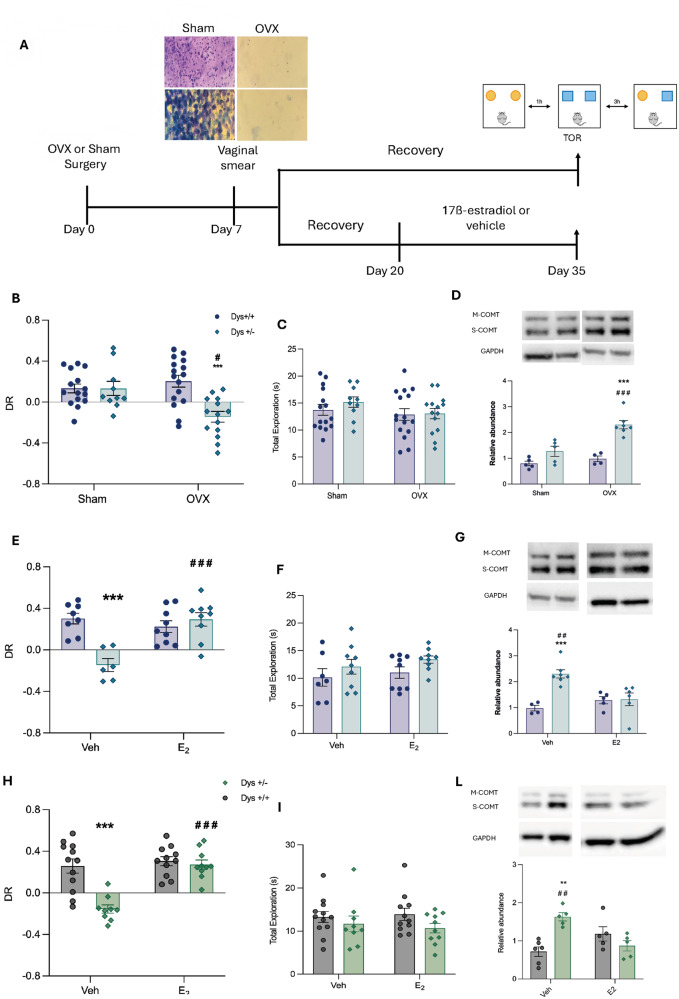
Ovariectomy produces cognitive impairments in female Dys + /− mice and 17ß-estradiol rescues Dys-dependent cognitive deficits in both Dys + /− ovariectomized female and Dys + /− male mice through the COMT contribution. **A** Timeline depicting the experimental procedure. **B** Temporal Order Recognition test. 2-way ANOVA analysis revealed a genotype effect F (1, 51) = 9.690, **p < 0.003, and a main effect of interaction surgeryXgenotype F (1,51) = 9.674, **p = 0.0031 but not a main surgery effect F (1, 51) = 3.425, *p* = 0.07. Bonferroni’s Multiple Comparison tests. *p = 0.011 Dys + /− Sham vs. Dys + /− OVX; ***p = 0.0001 Dys + /+ OVX vs Dys + /− OVX. **C** Total exploration in seconds. 2-way ANOVA, Genotype F (1,51) = 0.5846 *p* = 0.4480; Surgery F (1,51) = 2.06, *p* = 0.1573; SurgeryXGenotype F (1,51) = 0.3675, *p* = 0.547. Dys + /+ *n* = 31 (Sham *n* = 15, OVX *n* = 16), Dys + /− *n* = 25 (Sham *n* = 11, OVX *n* = 14). **D** COMT protein level alteration in the mPFC of Sham and OVX mice. 2-Way ANOVA revealed a main genotype effect F(1, 17) = 32.19 ***p < 0.0001, surgery effect F(1,17) = 14.67 **p = 0.0013 and a GenotypeXSurgery effect F(1, 17) = 7.225 *p = 0.0156. Bonferroni’s multiple comparison test ***p < 0.0001 vs Dys + /+ OVX, ^###^*p* = 0.0007 vs Dys + /− Sham (Dys + /+: OVX *n* = 4, Sham *n* = 5; Dys + /− OVX *n* = 7, Sham *n* = 5). **E** Temporal order recognition test in Veh or E2-treated OVX mice. 2Way-ANOVA analysis showed a Treatment effect F(1,30) = 11.73 **p = 0.0018 and a Genotype effect F(1,30) = 12.74 **p = 0.0012 as well as the interaction GenotypeXTreatment F(1,30) = 22.99 ***p < 0.0001. Bonferroni’s multiple comparisons test: ***p < 0.0001 Dys + /− Veh vs OVX, ***p < 0.0001 Dys + /+ Veh vs Dys + /− Veh. (Dys + /+ Veh *n* = 8, E2 *n* = 9; Dys + /− Veh *n* = 6, E2 *n* = 9). **F** Total exploration time. 2Way ANOVA showed no effects of E2 treatment in the total exploration time. 2Way-ANOVA Treatment effect F(1,30) = 0.8988 *p* = 0.3507, Genotype effect F(1,30) = 3.429 *p* = 0.0739, Interaction Genotype X Treatment F(1,30) = 0.0339 *p* = 0.8551. **G** COMT protein level alteration OVX Veh- and E2-treated female mice. 2-Way ANOVA revealed a main Genotype X Treatment Effect F(1, 18) = 11.54, **p = 0.0032 and a main genotype effect F(1, 18) = 12.82 **p = 0.0021 but not a main treatment effect F(1,18) = 3.184, *p* = 0.0912. Bonferroni multiple comparison test ***p = 0.0008 vs Dys + /+ Veh, ^##^*p* = 0.0044 vs Dys + /− E2 (Dys + /+: Veh *n* = 4, E2 *n* = 5; Dys + /− OVX *n* = 7, E2 *n* = 6). **H** Temporal order recognition test in Veh or E2-treated male mice. 2Way-ANOVA revealed a Genotype effect F(1,38) = 17.78 ***p = 0.0001 and a Treatment effect F(1,38) = 20.30 ***p < 0.0001 as well as an Interaction TreatmentXGenotype F(1,38) = 12.95 ***p = 0.0009. Bonferroni’s multiple comparisons test: ***p < 0.0001 Dys + /+ Veh vs. Dys + /− Veh, ***< 0.0001 Dys + /− Veh vs. Dys + /− E2 (Dys + /+ Veh *n* = 12, E2 *n* = 11; Dys + /− Veh *n* = 9, E2 *n* = 10). **I** Total exploration time in males. 2Way-ANOVA showed no effect of the treatment on the working memory performance: Treatment effect F(1,38) = 0.01954 *p* = 0.889, Genotype F(1,38) = 2.854 *p* = 0.0994, Interaction F(1,38) = 0.3202 *p* = 0.5748. All data are represented as mean ± S.e.m. **L** COMT protein level alteration in Veh and E2-treated male mice. 2-Way ANOVA revealed a main genotypeXtreatment effect F(1, 17) = 18.91, **p = 0.0004 and a main genotype effect F(1, 17) = 4.608, *p* = 0.0465 but not a main treatment effect F(1,17) = 1.129, *p* = 0.3029. Bonferroni’s multiple comparison test: **p < 0.001 vs Dys + /+ Veh, ^# #^*p* = 0.0097 vs Dys + /− E2 (Dys + /+: Veh *n* = 6, E2 n = 5; Dys + /− Veh *n* = 5, E2 *n* = 5).

To assess whether peripheral E2 levels were responsible for the lack of cognitive impairment in female Dys + /− mice, we used a surgical approach in which the ovaries, the main source of E2, were removed in both Dys + /+ and Dys + /− female mice (Fig. 2A). As expected, sham-operated female Dys + /− mice did not show differences compared to their Dys + /+ littermates when tested in the TOR test. Conversely, ovariectomized Dys + /− female mice exhibited poorer performance in the TOR test than Dys + /+ mice (Fig. 2B; 2Way-ANOVA: genotype effect F(1,51) = 9.690, **p < 0.003, surgery effect F(1,51) = 3.425, *p* = 0.07; Surgery-Genotype interaction F(1,51) = 9.674, **p = 0.0031 Bonferroni’s Multiple Comparison test. *p = 0.011 Dys + /− Sham vs. Dys + /− OVX; ***p = 0.0001 Dys + /+ OVX vs Dys + /− OVX; Dys + /+ Sham *n* = 15, OVX *n* = 16, Dys + /− Sham *n* = 11, OVX *n* = 14). This suggests that the loss of estrogens recapitulates the behavioral phenotype of male Dys + /− mice.

Rodents that underwent ovariectomy have been widely reported to show different characteristics, such as increased body weight and reduced uterine weight due to uterus involution [66, 67]. To validate the surgical approach of ovariectomy used in these experiments, the body weight of the mice was monitored from the immediate postoperative period (two weeks after ovariectomy) for 15 days, and the uterine weight was measured immediately after sacrifice. The 15-day analysis showed a main effect of surgery with a statistically significant increase in body weight (BW) in both Dys + /+ and Dys + /− OVX compared to the respective sham-operated animals (Fig. S1D; 3Way-ANOVA, Day F(7,273) = 4.349, ***p = 0.0001; Surgery F(1,39) = 28.88, ***p < 0.001 and genotype F(1,39) = 20.50 **p < 0.0001 Day-Genotype interaction F(7,273) = 3.730 *p* = 0.0007. Fisher’s LSD Multiple comparison: ***p < 0.001, **p < 0.01 vs. Dys + /+ Sham, *p < 0.05, **p < 0.01, ***p < 0.001 vs. Dys + /− Sham). Uterine weight (UW), assessed after sacrifice, shows a main effect of the surgery procedure demonstrated by the reduced %UW/BW ratio in OVX compared to sham (Fig. S1E; 2Way-ANOVA Surgery effect F(1,27) = 65.16,***p < 0.0001, Genotype effect F(1,27) = 0.02435 p = 0.8772, Surgery-Genotype interaction F(1,27) = 0.00061 *p* = 0.9804. Fisher’s LSD Multiple Comparisons: ***p < 0.001 vs Dys + /+ sham,***p < 0.001 vs Dys + /− sham).

These results suggest that genetic variants reducing Dys expression and affecting cognitive abilities could become functional in females only when estrogen’s protective effect is attenuated.

### 17ß-estradiol rescues Dys-dependent cognitive deficits in both Dys + /− male and Dys + /− ovariectomized female mice

To identify the role of E2 in Dys-dependent cognitive functions, we tested Dys + /− male and female mice in the TOR test. We achieved the control over the hormonal status of Dys female mice using ovariectomy (OVX) and pharmacological treatment with exogenous E2. Therefore, we first ovariectomized Dys + /− female mice, in order to have two cohorts of animals, male and female, with both the same behavioral performance in the TOR test and a similar E2 hormonal state. Then, we treated both Dys + /− male and Dys + /− OVX female mice with E2 administered peroral for 15 days (Fig. 2A). Lastly, we tested treated Dys + /− male and Dys + /− OVX female mice in the TOR behavioral paradigm 24 h after the last administration. The 2way-ANOVA revealed the presence of a significant Treatment-Genotype interaction. Chronic E2 supplementation rescued cognitive impairments in Dys + /− OVX female mice without affecting the cognitive performance of controls (female Dys + /+ OVX mice; Fig. 2E). These data suggest that peripheral estrogen fluctuations throughout the lifespan might concur to the magnitude of the heritable component of cognitive dysfunctions in female patients with schizophrenia.

To determine whether exogenous E2 treatment is able to rescue Dys-related cognitive dysfunction in males, we chronically treated Dys + /− and Dys + /+ male mice with E2 for 15 days. Estradiol supplementation rescued cognitive impairments exhibited by Dys + /− male mice, without influencing the performance of controls (Dys + /+ male mice; Fig. 2H). In both male and female mice, E2 treatment had no consequences on total exploration time (Fig. 2F–I).

### Catechol-O-Methyltransferase (COMT) contributes to the sexual dimorphism in dysbindin-related cognitive functions

Western Blot analysis carried out on mPFC of Dys + /− mutant and control mice (Dys + /+) showed modulation of COMT protein expression according to E2 levels (OVX or E2 treatment; Fig. 2D–G–L). In basal conditions, Dys + /− females showed no differences in COMT levels in mPFC as compared with their controls (Dys + /+;p = 0.144; Fig. 2D). The lack of E2 in OVX female animals increased COMT levels in Dys + /− but not in their controls (sham operated Dys + /− and Dys + /+; 2Way-ANOVA Genotype effect F(1,15) = 25.49 ***p = 0.0001, Surgery effect F(1,15) = 7.854 *p = 0.0134. Bonferroni multiple comparison test ***p < 0.0001 vs Dys + /+ OVX, ^#^*p* < 0.05 vs Dys + /− Sham. Figure 2D). Notably, the chronic treatment with E2 restored COMT to control levels in OVX Dys + /− female mice, confirming the presence of a Treatment-Genotype interaction in the modulation of COMT protein (2Way-ANOVA Genotype-Treatment interaction F(1,16) = 7.898, *p = 0.0126, Genotype effect F(1,16) = 9.678 **p = 0.001, Treatment effect F(1,16) = 1.855, *p* = 0.912. Bonferroni’s multiple comparison test **p < 0.0001 vs Dys + /+ OVX, #p < 0.05 vs Dys + /− E2. Fig. 2G). These data indicate that E2 selectively modulates COMT levels in the mPFC of female animals with disrupted dopamine transmission triggered by Dys reduction. The higher COMT levels in mPFC of Dys + /− male mice in basal conditions (Fig. 2L) confirm that the COMT-Dys functional interaction is involved in the modulation of cognitive functions. Consistent with these results, chronic treatment with E2 is capable of restoring the COMT levels of Dys + /− male mice to the control levels (Fig. 2L), suggesting the presence of a sex-driven Dys-COMT epistatic interaction in the control of cognitive functions in mice.

### Dysbindin gene expression changes in a sex-dependent manner in humans

**Fig. 3:**
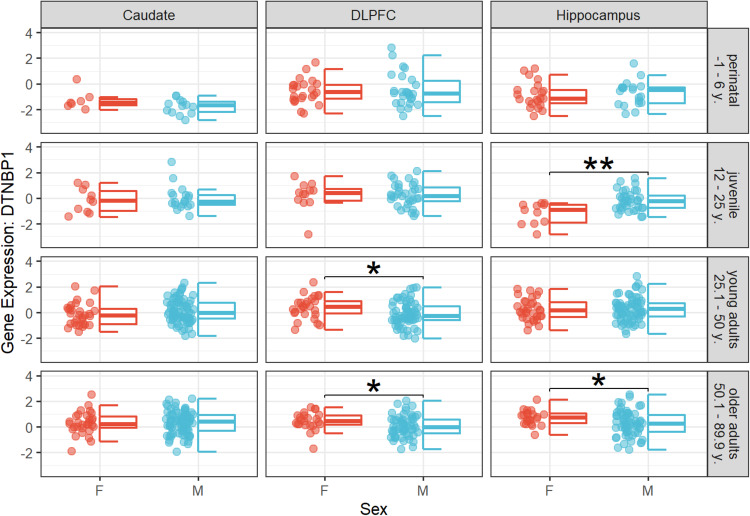
Sex differences of DTNBP1 gene expression in samples separately for age groups, genes, and brain regions. Box plots showing differences in terms of gene expression quantified in three brain regions, i.e., caudate, DLPFC, and hippocampus, during the perinatal period (up to the age of 6 years), juvenile period (between 12–25 years of age), younger adulthood (25-50 years of age), and older adulthood (above 50 years of age). Separate two-tailed Welch two-sample t-tests revealed significantly higher gene expression in males compared to females during the juvenile period for DTNBP1 in the hippocampus, t(15) = 3.48, *p_FDR_ = 0.010. In adults significantly higher gene expression was found in females compared with males; namely greater DTNBP1 expression in the DLPFC in younger, t(51) = −2.37, p_FDR_ = 0.065, and older adults, t(49) = −2.19, *p_FDR_ = 0.050, and higher DTNBP1 expression in the hippocampus in older adults, t(62) = −2.56, *p_FDR_ = 0.038. No other significant sex differences have been reported, all p_uncorr_ > 0.05. Abbreviations: F female, M male, y years of age, DLPFC dorsolateral prefrontal cortex.

To discern variations in Dys gene expression between sexes, we tested sex differences in *DTNBP1* expression in the hippocampus, DLPFC, and caudate through two-sample t tests accounting for regional specificity of gene expression across age groups, i.e., perinatal (1–6 years), juvenile (12–25 years), young adults (25.1–50 years), and older adults (50.1–90 years). We found significantly higher gene expression in males compared with females during the juvenile period for *DTNBP1* in the hippocampus, t(15) = 3.48, *p_FDR_ = 0.010. After the juvenile period, significantly higher gene expression was found in women compared with men; namely, nominally significantly higher expression of *DTNBP1* in DLPFC in younger adults, t(51) = −2.37, *p_FDR_ = 0.065, and older adults, t(49) = −2.19, *p_FDR_ = 0.050, and greater expression of *DTNBP1* in the hippocampus in older adults, t(62) = −2.56, *p_FDR_ = 0.038. Results are depicted in Fig. 3. These results indicate the existence of sex- and age-related differences in Dys expression, suggesting a potential sex-biased effect on Dys-dependent functional outcomes. Chi-square tests to assess sex and age differences across postmortem samples revealed a significant sex difference across age groups specifically for hippocampus samples, χ^2^(3,276) = 10.90, *p* = 0.012, whereas no significant relationship between age group and sex was found for DLPFC, χ^2^(3, 261) = 6.14, *p* = 0.105, or caudate, χ^2^(3, 259) = 0.74, *p* = 0.863. The results of these tests exclude possible sampling bias effects from influencing our results regarding DLPFC and caudate. However, they also caution about potential sampling bias effects of results in the hippocampus.

### Interactions between Catechol-O-Methyltransferase single nucleotide polymorphism rs4680 and sex on DTNBP1 gene expression in humans

**Fig. 4:**
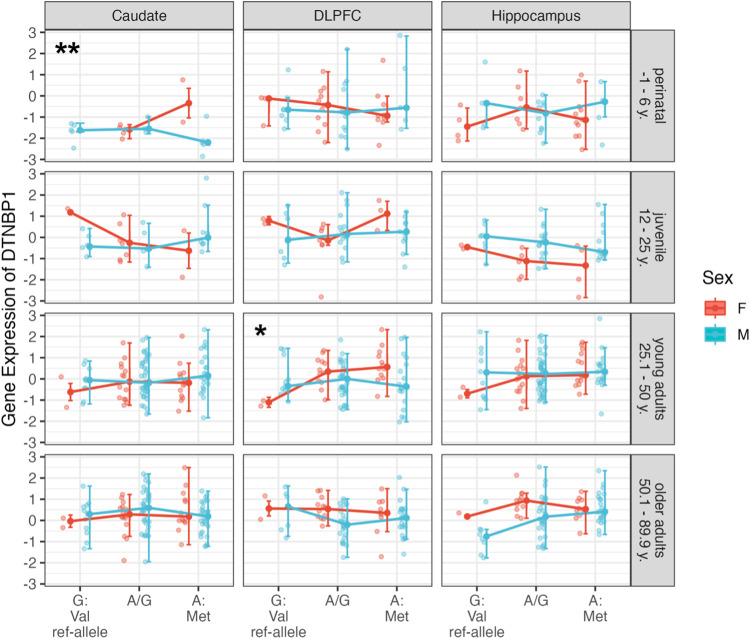
Interactions between Catechol-O-Methyltransferase single nucleotide polymorphism rs4680 and sex on DTNBP1 gene expression separately for age groups and brain regions. Error bars showing the interactions resulted from separate two-way analyses of variance between COMT and sex on DTNBP1 gene expression across age groups, that is, the perinatal period (up to the age of 6 years), juvenile period (between 12 and 25 years of age), younger adulthood (25-50 years of age), and the older adulthood (above 50 years of age) and separately for caudate, dorsolateral prefrontal cortex and hippocampus. Significant interactions have been found between sex and the COMT rs4680 on DTNBP1 expression for DLPFC in young adults (F(2,87) = 3.34, *p_uncorr_ = 0.040), and caudate in the perinatal age group (F(1,19) = 10.22, **p_uncorr_ = 0.006). All other analyses of variance yielded no significant interaction effects between sex and COMT rs4680 on DTNBP1 expression, all p_uncorr_ > 0.05 Abbreviations: F female, M male, y. age in years, DLPFC dorsolateral prefrontal cortex.

To replicate the results observed in mice on the interaction between Dys gene expression and COMT activity in human *postmortem* samples, we separately tested for age groups and brain regions for significant interaction between sex and the *COMT rs4680* genotype on *DTNBP1* expression using 2way-ANOVAs. We found a significant interaction between sex and *COMT rs4680* on *DTNBP1* expression in the DLPFC in young adults (F(2,87) = 3.34, *p_uncorr_ = 0.040), and in the caudate in the perinatal - age group (F(1,19) = 10.22, **p_uncorr_ = 0.006; Fig. 4). All other analyses of variance yielded no significant interaction effects between sex and *COMT rs4680* genotype on *DTNBP1* expression, all p_uncorr_ > 0.05 (Fig. 4). These findings confirm a functional interaction of COMT on Dys gene expression across sexes, specifically evident in the DLPFC during adulthood.

### Sex-driven Dys-COMT epistatic interaction in fMRI brain activity during the N-back task performance

To establish a relationship between the observed interaction of *COMT* on Dys gene expression across sexes in *postmortem* DLPFC samples and its reflection on behavioral outcomes, mirroring the results observed in mice, we analyzed brain activity in a sample of living humans while performing the N-back task – a task sensitive to dopaminergic signaling in DLPFC. The two-sample t-test revealed a significant difference between the males and females included in the sample in terms of age, with males older than females (t(206) = 2.07, *p* = 0.04). Furthermore, separate two-sample t-tests revealed significantly higher IQ (t(160) = −4.71, *p* = 4.63 × 10^−^^06^), and higher working memory performance in terms of Hit rate (t(131) = −4.30, *p* = 2.64 × 10^−^^05^), working memory efficiency rate, i.e., the ratio between accuracy and reaction time (t(101) = −4.21, *p* = 4 × 10^−^^05^) [57], and lower reaction time (t(104) = 4.61, *p* = 7.18 × 10^−^^06^) in males compared with females. To control for these differences, we included age and IQ as nuisance covariates in group statistical analysis, while interindividual variability during the N-back task was used to test brain-behavior associations. Further control analysis on age-related effects is reported in the Supplementary Information, Section 2.7.

**Fig. 5:**
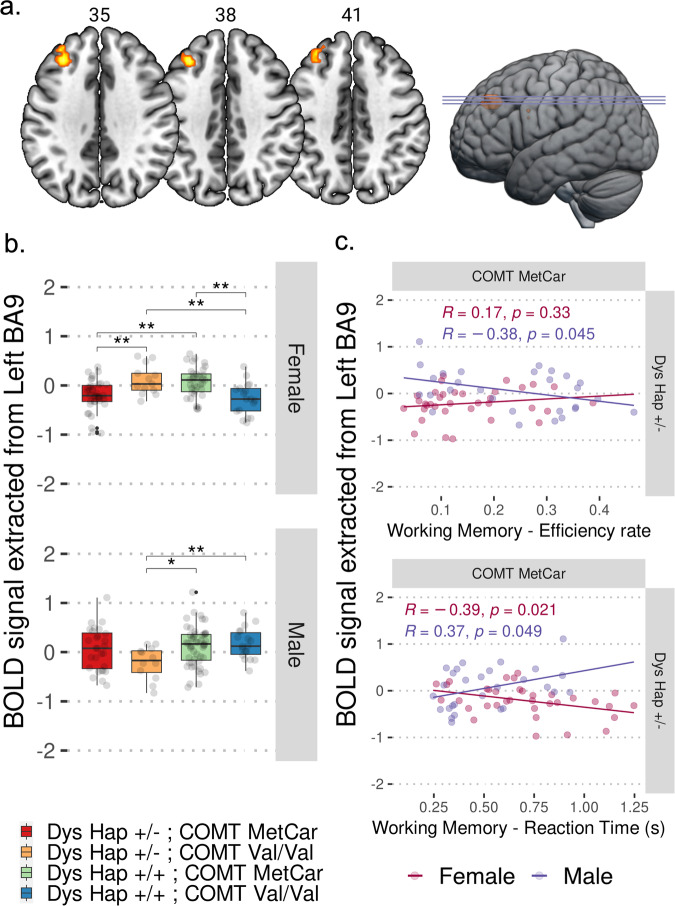
Interaction between COMT rs4633, Dysbindin Hap, and sex on brain activity during the N-back task performance. **a** Brain multi-slice sections and rendering showing the significant DLPFC activity resulting from the three-way interaction between COMT genotype, Dys Hap, and sex during N-Back task located in the left DLPFC (Brodmann Area 9; MNI coordinates x = −30, y = 32, z = 38; k = 52, Z = 3.49; *p_TFCE-FWE_ = 0.04). The color bar indicates log_10_ TFCE scores ranging from 1.3 to 4 as suggested by Smith and Nichols [66]. **b** Box plots showing the differences between the COMT genotype and Dys Hap groups in the female and male groups assessed by the two-sample t test and corrected for multiple comparisons (k = 6; p_FDR_ < 0.05). The BOLD estimates extracted from Brodmann Area 9 were significantly higher in COMT^Val/Val^ females with Dys Hap +/− compared with Dys Hap +/+ (t(27) = 3.37; **p_FDR_ = 0.008), while COMT^MetCar^ females with Dys Hap +/− presented lower BOLD estimates compared with Dys Hap +/+ (t(69) = −3.68; **p_FDR_ = 0.005). Furthermore, females with Dys Hap +/− and COMT^Val/Val^ presented higher estimates than COMT^MetCar^ (t(23) = 3.22; **p_FDR_ = 0.009), as well as females with Dys Hap +/+ and COMT^MetCar^ presented higher estimates than COMT^Val/Val^ (t(32) = 3.63; **p_FDR_ = 0.005). Also, the BOLD estimates extracted from BA9 were different in COMT^Val/Val^ males with Dys Hap +/− compared with Dys Hap +/+ (t(23) = −3.21; **p_FDR_ = 0.004) in the opposite direction compared with the female analysis, as well as males with Dys Hap +/− and COMT^Val/Val^ presented trending lower estimates than COMT^MetCar^ (t(27) = −2.19; p_FDR_ = 0.06). Only significant differences corrected for multiple comparisons have been reported in the figure (p_FDR_< 0.05). **c** Scatterplots showing the association between brain activity estimates extracted from left Brodmann Area 9 and behavioral indices from the N-back task, i.e., the efficiency rate and the reaction time (in seconds). General linear models revealed a significant negative association between the reaction time and brain activity estimates extracted from left Brodmann Area 9 in females with the COMT^MetCar^ and Dys + /− (r = −0.39; p_uncorr_ = 0.02), while a significant positive association between the reaction time and activity estimates (r = 0.37; p_uncorr_ = 0.05), and a negative association between the efficiency rate and activity estimates (r = −0.38; p_uncorr_ = 0.04) were found in males. All regression’s r and nominal p-values are reported in the figure. BOLD Blood Oxygen Level Dependent, BA9 Brodmann Area 9.

To investigate the interaction between COMT genotype, Dys Hap, and sex (categorical predictors: male vs female; COMT^Val/Val^ vs COMT^MetCar^; Dys Hap + /+ vs Dys Hap + /−), we performed a voxel-wise three-way ANOVA on individual brain activity during the N-back task. The main effects of the sex, COMT genotype, and Dys Hap were not significant (p_TFCE-FWE_ > 0.05). Instead, a significant three-way interaction between sex, COMT genotype, and Dys Hap in a cluster located in the left DLPFC (Brodmann Area 9; Z = 3.49; *p_TFCE-FWE_ = 0.04; Fig. 5a) suggested a sex-dependent functional epistasis between COMT and Dys Hap, in absence of detectable main effects.

To further explore this interaction in the DLPFC activity, we performed *post hoc* two-sample t-tests comparing DLPFC activity on all combinations of *COMT* genotypes and Dys Hap within the sex groups. The results in females showed a reversed activation pattern as function of Dys Hap comparing COMT^Val/Val^ with higher DLPFC activity than COMT^MetCar^ in Dys + /− condition (t(23) = 3.22; **p_FDR_ = 0.009), and COMT^Val/Val^ with lower DLPFC activity than COMT^MetCar^ in Dys + /+ condition (t(32) = 3.63; **p_FDR_ = 0.005). On the other hand, results in males showed no significant differences comparing COMT^Val/Val^ and COMT^MetCar^ as a function of Dys Hap, however, DLPFC activity was lower in COMT^Val/Val^ males with Dys Hap + /− compared with Dys Hap + /+ (t(23) = −3.21; **p_FDR_ = 0.009) in the opposite direction compared with the females’ activation pattern (t(27) = 3.37; **p_FDR_ = 0.008). Aligned with the findings in the mouse model, our results indicate that the Dys Hap affects cognitive brain function in a sex-biased manner, interacting with the COMT rs4633 genotype. Specifically, Dys Hap + /− males show reduced DLPFC activity in the presence of possibly higher COMT levels, whereas Dys Hap + /− females show increased DLPFC activity.

Regarding functional behavioral outcomes, we conducted linear regressions to assess the relationship between individual DLPFC activity estimates and working memory performance across sex groups. We separately testes the hit rate, reaction time, and the efficiency rate based on the N-back task responses including the *COMT* rs4633 genotype, and Dys Hap as categorical factors. Linear models revealed a significant negative association between the reaction time and DLPFC activity estimates in females with COMT^MetCar^ and Dys + /− (*r* = −0.39; p_uncorr_ = 0.02), while a significant positive association between the reaction time and the DLPFC activity estimates (*r* = 0.37; p_uncorr_ = 0.05), and a negative association between the efficiency rate and the DLPFC activity estimates (r = −0.38; p_uncorr_ = 0.04) were found in males with COMT^MetCar^ and Dys Hap + /−. Results are depicted in Fig. 5c and Fig. S3.

Taken together these findings indicate that DLPFC activity exhibits sex-based variations and is contingent upon the genetic combination of Dys Hap and COMT. This variability potentially influences individual cognitive performance, which we found lower in males with Dys +/− and elevated COMT levels, while it was preserved in females under similar conditions.

## Discussion

This study aimed to investigate sex differences in dysbindin-related cognitive dysfunctions with relevance to pathophysiological mechanisms of sexual dimorphisms in schizophrenia. Through a multidisciplinary approach combining behavioral and molecular findings on genetic animal models and human genetic and fMRI data, our results revealed a sexual dimorphism in working memory performance associated with a functional genetic variant characterized by reduced Dys protein expression, through the functional epistatic contribution of COMT (as recapitulated in Fig. S5). Previous evidence demonstrated that genetic-dependent reduced expression of Dys exerts a prominent effect on cognitive-related PFC functional activation in humans and mice [15, 38]. Nevertheless, these studies did not consider potential gene-by-sex interactions. However, many human diseases, including schizophrenia, exhibit sex-specific characteristics, including age of onset, prevalence, progression, severity of symptoms, and response to treatment [68, 69]. Different factors may contribute to these sex-dependent features, including endogenous [5, 70], exogenous [71], and genetic factors [72]. Endogenous factors, such as hormones or their alteration, could trigger sex-specific regulatory pathways that influence molecular traits and contribute to sex differences in the genetic architecture of complex diseases [73]. The approaches used in this scenario, wherein a functional genetic variant changes the relative expression of the *DTNBP1* gene in a sex-dependent manner combined with a preclinical model of ovariectomy in female Dys mutant mice, allowed us to distinguish phenotypes regulated by sex-by-gene interaction from phenotypes for which Dys exerts independent or no effects. Furthermore, consistent findings in humans and mice strengthen the conclusion that reduction of Dys activity causes cognitive impairments only in males [15]. Intriguingly, our findings suggest that genetic variants that reduce the expression of Dys protein could be disadvantageous in females when the protective effect of estrogens decreases with aging, i.e., during menopause [30].

The quantification of *DTNBP1* gene expression in human post-mortem samples revealed the presence of sex-driven and age-related changes in Dys expression. Interestingly, *DTNBP1* expression is differentially modulated in males and females throughout life, with an increase in Dys expression in the DLPFC of females after the juvenile and adulthood periods. Thus, parsing the age of the sample is important in sex studies of gene expression to account for hormonal changes during the lifespan. Consistent with these results, the findings from mutant mice with reduced levels of Dys showed the presence of a sexual-behavioral dimorphism driven by the *DTNBP1* gene. Notably, we found the effect of the estrous cycle to be critical to female mice cognition, showing a working memory impairment associated with lower Dys expression and estrogen deficiency induced by ovariectomy. These findings are supported by earlier studies showing that working memory and cognitive function, in human and mouse females, are highly dependent on the physiological estrogen fluctuation throughout the estrous cycle [60–64, 74]. Interestingly, the effects of the estrous cycle were more prominent in mPFC-driven cognitive function, whereas only a marginal role of sex hormones was observed in other schizophrenia-relevant phenotypes. These findings corroborate prior evidence revealing estrogen’s beneficial effects on frontal cortex-dependent tasks in mice [75] and humans [76]. The impact of estrogen deprivation in mice or physiological decline in women is widely associated with cognitive performance decline [77]. The loss of the main source of estrogens in female Dys mice led to an impairment in working memory similar to that shown by male Dys mice tested on the same mPFC-dependent behavioral task. Furthermore, chronic treatment with E2 also rescued Dys-related cognitive dysfunction in male Dys + /− mice. Considering the high haplotype frequency of the genetic variants of Dys within the general population (ranging from 0.58 to 0.95) and among patients with schizophrenia (ranging from 0.59 to 0.95), as reported in the latest Genome-Wide Association Study on schizophrenia [78], we speculate that further studies of E2 might consider it as an add-on therapy for patients with schizophrenia [79]. Furthermore, estrogens can have also beneficial pharmacokinetic effects as they directly increase the plasma concentration of antipsychotic drugs by regulating enzymes that metabolize antipsychotics, which is most evident for clozapine and olanzapine [80].

The presence of an indirect interaction between estrogens and Dys, mediated via COMT is in line with studies showing that the COMT gene contains two ERE sequences [29], and the data demonstrating the central inhibitory effect of catechol estrogen on COMT activity [81]. Overall, our findings in mice provide evidence that the beneficial cognitive effects of E2 should be considered in combination with the epistatic interaction of COMT/Dys genes. Notably, neuroprotective effects of augmented estrogen levels have been reported in post-menopause women [63, 72], as well as in men undergoing the gender-affirming hormone treatment, combined therapy of estrogen and antiandrogen hormones, resulting in enhanced cognitive abilities [74, 82]. The analysis of *COMT rs4680* and sex on *DTNBP1* gene expression in humans further corroborates our hypothesis. We showed an interaction between sex and *COMT* on *DTNBP1* expression in the DLPFC selectively in the younger adults between 25 and 50 years of age when individuals reach full maturity. This study adds to previous knowledge showing the implication of *COMT rs4680* in schizophrenia-related cognitive impairments, its relationship with sex, and Dys gene expression in humans during adulthood [72].

The assessment of DLPFC functional activity in humans using the fMRI further reinforces our findings and suggests an approach to translate neurobiological mechanisms from mice to humans. However, further investigation in larger and longitudinal samples would be needed to disentangle the role of estrogen levels on cognition in interaction with Dys/COMT. Indeed, the unavailability of estrogen information in our female human samples precludes a comprehensive understanding of how estrogens might influence cognitive processes associated with Dys expression variability and COMT in humans. Moreover, as our brain imaging sample included only adults, we could not explore potential developmental and aging effects in humans [83]. While it is possible that males and females differ in the age component of the functional interaction we detected, when age differences between groups were corrected the interaction persisted. This prompts speculation that studies on schizophrenia that mainly combine male and female adults to investigate potential etiological aspects may have overlooked molecular effects associated with sex and age.

In conclusion, the present study supports the relevance of sexual dimorphism on the cognitive mechanisms associated with schizophrenia, and how this could be affected by age-specific changes. These findings can provide new insights into the etiology and treatment of psychiatric disorders and contribute to the development of additional tools for patient stratification for precision medicine.

## Supplementary information

Supplementary Information

## Data Availability

The data that support the findings of this study are available from the corresponding author, upon reasonable request. Post-mortem data that support the findings were used under license from the Lieber Institute for Brain Development (LIBD). Data are, however, available from the authors upon reasonable request with detailed motives and objectives. Participant data reported in this article can be shared in compliance with current data protection regulations by the European Union. Data requestor will be required to sign a data access agreement with the authors and the University of Bari.
