# Supplementary material for: Sex dimorphism controls dysbindin-related cognitive dysfunctions in mice and humans with the contribution of COMT
Source: Mol Psychiatry. 2024 Mar 26;29(9):2666–77. doi: 10.1038/s41380-024-02527-3 (PMC11420087; doi:10.1038/s41380-024-02527-3)
Supplement: Supplementary file 1 — Supplementary Information [file 41380_2024_2527_MOESM1_ESM.docx]

**Supplementary Information**

**Sex dimorphism controls dysbindin-related cognitive dysfunctions in mice and humans with the contribution of COMT**

Federica Geraci^1#^, Roberta Passiatore^2,3,4#^, Nora Penzel^2^, Samuele Laudani^1^, Alessandro Bertolino^2,5^, Giuseppe Blasi^2,5^, Adriana C.E. Graziano^1^, Gianluca C. Kikidis^2,4^, Ciro Mazza^2^, Madhur Parihar^5^, Antonio Rampino^2,5^, Leonardo Sportelli^2,4,6^, Nicolò Trevisan^7^, Filippo Drago^1^, Francesco Papaleo^8^, Fabio Sambataro^7‡^, Giulio Pergola^2,4,9‡^ and Gian Marco Leggio^1‡*^

^1^ Department of Biomedical and Biotechnological Sciences, University of Catania, 95123 Catania, Italy

^2^ Department of Translational Biomedicine and Neuroscience, University of Bari Aldo Moro, 70124 Bari, Italy

^3^ Institute of Neuroscience and Medicine, Brain & Behavior (INM-7), Research Centre Jülich, 52428 Jülich, Germany

^4^ Lieber Institute for Brain Development, Johns Hopkins Medical Campus, 21205 Baltimore, MD, United States

^5^ Psychiatric Unit - University Hospital, 70124 Bari, Italy

^6^ Department of Human Genetics, Radboud University Nijmegen, 6525 GD Nijmegen, The Netherlands

^7^ Department of Neuroscience (DNS), University of Padova, 35121 Padova, Italy

^8^ Genetics of Cognition Laboratory, Neuroscience area, Istituto Italiano di Tecnologia, Genova, Italy.

^9^ Department of Psychiatry and Behavioral Sciences, Johns Hopkins University School of Medicine, 21205 Baltimore, MD, USA

^#^ These authors have contributed equally to this work

^‡^ These authors shared the last authorship

* Corresponding author:

Gian Marco Leggio, Ph.D.

Department of Biomedical and Biotechnological Sciences, University of Catania,

Via Santa Sofia 97, 95123 Catania, Italy.

Tel.: +39 095 478 1200

E-mail: gianmarco.leggio@unict.it

**METHODS**

# ANIMALS

Animal sample size was chosen based on studies using related methods and is similar to what is generally employed in the field. Randomization was not used to assign animals to experimental groups, and the investigator was blinded to the genotype of animals. All experiments were carried out according to EU Directive 2010/63/EU and the Institutional Animal Care and Use Committees of Catania University.

## Drugs

For each 30g mouse, 1.12µg of 17ß-estradiol was dissolved in 0.312 µl of sesame oil (Sigma-Aldrich, St. Louis, MO, United States) and then mixed with 60 mg of Nutella®. Control animals received the identical mix except for 17ß-estradiol. The animals were trained to eat hormone-free Nutella for five days prior to the experimental procedure. For the first three days, mice were trained in their home cages (group housed), and on the fourth and fifth days, animals were habituated in separate cages (single housed) to mimic the experimental procedure. The mice were placed in separate cages for each feeding occasion during the experiment. Body weight was routinely measured.

## Estrous cycle phases identification

Handled female mice were placed on the cage lid and held by the tail. Cells were collected via vaginal lavage with 1% PBS and immediately applied to a glass slide.

Samples were air-dried and stained with Toluidine Blue (Sigma-Aldrich, St. Louis, MO, United States). The samples were observed using a light microscope, and the estrous cycle stages were identified as previously described ^1^. Proestrus is defined by mostly nucleated epithelial cells; estrus is defined by mostly cornified epithelial cells; metestrus is characterized by cornified epithelial cells and leukocytes, and mainly leukocytes represent diestrus.

## Temporal order recognition test

The animals were explored in an evenly illuminated (9±1 lux) square open field (40x40x40 cm, Ugo Basile, Gemonio, Italy), with the floor covered with sawdust. The objects presented (Duplo blocks, Lego®) were different in shape, color, and size and too heavy to move. The test consisted of two sample phases and a test trial. In both sample phases, mice were allowed to explore two identical objects for 5 minutes. Different objects were used for sample phases 1 and 2, with a delay between sample phases of 1h. During the test trial, carried out three hours after sample phase 2, the mouse was allowed to explore a copy of the object from sample phase 1 and sample phase 2. The objects were cleaned after each phase with a 10% ethanol solution to avoid olfactory cues. Temporal memory was considered intact if the animals spent more time exploring the less recent object (sample phase 1) than the more recent object (sample phase 2). Animals that did not reach a minimum of 2 seconds of exploration in any phase were excluded from the analysis. Data are expressed as discrimination ratio (DR) calculated as (time exploring the less recent object - time exploring the more recent object/total exploration time).

## Protein extraction and Western Blot analysis

The animals were sacrificed by cervical dislocation, and mPFC samples were collected and frozen on snap in dry ice. According to the manufacturer's instructions, proteins were extracted using the RNA/DNA/Protein Purification Plus Kit (Norgen Biotek, Canada). COMT protein expression was evaluated by Western Blot analysis. Twenty micrograms of total protein in the supernatant were loaded on each lane and separated by 4-12% Novex Bis-Tris gel electrophoresis (NuPAGE, Invitrogen, USA). Proteins were transferred to nitrocellulose membranes (Invitrogen, USA) in a wet system. The transfer of proteins was verified by staining the nitrocellulose membranes with Ponceau S. Membranes were blocked using iBind Solution Kit (Invitrogen, USA) and incubated with primary antibodies for COMT (1:2000 dilution, #6119770, mouse mAb, Clone 4/COMT/RUO, BD Biosciences, Franklin Lakes, NJ) and GAPDH-HRP (1:1000 dilution). Antibodies were diluted using the iBind Solution Kit (Invitrogen, USA). COMT was detected with horseradish peroxidase-conjugated secondary antibody (1:1000 dilution, according to the manufacturer's instructions; iBind Flex, Invitrogen, USA) using enhanced chemiluminescence detection Supersignal West Pico Chemiluminescent Substrate (Pierce Chemical Co., Rockford, IL). The relative density of the protein bands was normalized to the levels of GAPDH. Immunoblot bands were quantified using ImageJ software for gel densitometry (provided in the public domain by the National Institute of Health, available online: <https://imagej.nih.gov/ij/download.html>). Data are expressed as the relative abundance corresponding to signal intensity.

## Statistical analysis

All data assume a normal distribution that has been assessed by D'Agostino-Pearson's "omnibus K2" normality test. A Levene’s test was also applied to verify equality of variances. Data were subjected to parametric tests, one-way ANOVA, and two-way ANOVA with repeated measures when appropriate.

For all data analyses, differences among individual means were assessed using Bonferroni post hoc tests. Body weight and uterus weight were analyzed using Fisher's Least Significant Difference (LSD) test. The IQR method was employed to identify outliers. Differences were considered statistically significant when p < 0.05. The dispersion estimate is the standard error of the mean (s.e.m.). All data are presented as means ± s.e.m.

# HUMANS

## Regional co-expression quantification and genotyping

Reads Per Kilobase per Million mapped reads (RPKMs) quantified gene-level messenger-RNA expression and separately annotated total gene expression for each brain region ^2^ using GENCODE release 25 (GRCh38.p7, https://www.gencodegenes.org). Genes entered further processing if they had a median RPKM ≥ 0.1 and were free of floor effects (maximum 20% of zeroes per gene). We log-transformed RPKM values with an offset of 1, i.e., log_2_(RPKM+1). To investigate biologically meaningful age groups, we divided the sample into four different groups, i.e., a perinatal group (up to the age of six years), juveniles (between 12-25 years of age), younger adults (25-50 years of age), and older adults (above 50 years of age), as previously reported ^3^.

The inter-array correlation identified outlier subjects deviating more than three standard deviations from the mean ^4^ relative to their age period. To reduce the risk of removing age-related signals, we regressed out the effect of the following variables from each brain region cohort separately: mitochondrial mapping rate, rRNA rate, gene mapping rate, RNA Integrity Number, ancestry estimated via the first ten genomic eigenvariates as computed in Collado-Torres, Burke ^2^, and estimated individual neuronal proportion – the latter confounder was included to obtain an appropriate comparison between subjects of different age. Rank-normalizing of the residuals of each gene using the *blom* function^5-8^ was employed to limit the impact of deviations from normality in the expression data ^9^.

Genotype data were processed as previously described ^10^. First, genotype imputation was performed on high-quality observed genotypes (removing low-quality and rare variants) using the TOPMed imputation server pipeline ^11^: phasing was performed with Eagle V2.4 (https://alkesgroup.broadinstitute.org/Eagle/), and imputation with minimac4 using HRC r1.1 2016 as the reference panel (genome build hg19) ^12^. We retained common variants (MAF >1%) with high imputation quality (INFO >0.9) that were present in the majority of samples (missingness < 10%) and were in Hardy Weinberg equilibrium (at p > 1×10^−6^) using the Plink tool kit version 2 ^13^.

## Inclusion/exclusion criteria for the human fMRI sample

All participants were assessed in person with a Structured Clinical Interview for the Diagnostic and Statistical Manual of Mental Disorders Version IV (DSM IV). Exclusion criteria included the presence of a DSM IV Axis I diagnosis at the time of the study and by history, having a first-degree relative with a psychiatric disorder, IQ < 80, recent drug or alcohol abuse (within one year), or >5 years of previous abuse, current psychotropic pharmacological treatment, history of head trauma with loss of consciousness, and metal implants. The local ethics committee approved the experimental protocol. All subjects received a complete description of the study and its procedures. According to the Declaration of Helsinki, written informed consent was obtained after fully understanding the protocol.

## Genotype determination in the human fMRI sample

Participants underwent blood withdrawal for subsequent DNA extraction from peripheral blood mononuclear cells. To this aim, approximately 20ml of fresh blood was obtained through a conventional venous blood collection with 10ml EDTA Vacutainer Venous Blood Collection Glass Tubes (Vacutainer ®). Approximately 200 ng of DNA was used for genotyping analysis. DNA was concentrated at 50ng/µl (diluted in 10 mM Tris/1mM EDTA) with a Nanodrop Spectrophotometer (ND-1000). Samples were genotyped using various Illumina Bead Chips, including 510K/610K/660K/2.5M.

Quality control was performed using PLINK (version 1.07; http://pngu.mgh.harvard.edu/purcell/plink/) ^13^ accounting for SNP missingness < 0.05 (before sample removal); subject missingness < 0.02; autosomal heterozygosity deviation (|Fhet| < 0.2); SNP missingness <0.02 (after sample removal), SNP Hardy-Weinberg equilibrium (HWE: P>10^−6^) and minor allele frequency (MAF) >0.01. Genotype imputation was performed using the pre-phasing/imputation stepwise approach implemented in IMPUTE2 / SHAPEIT (chunk size of 3 Mb and default parameters) and Phase 3 1000 genome as reference panel ^14, 15^. After imputation, we considered imputed dosage data for each SNP with imputation quality (INFO) > 0.9. The COMT Val158Met SNP, rs4680, was not available in our sample; thus, we selected a Linkage Disequilibrium (LD)-SNP named rs4633 (D' = 0.996, R^2^ = 0.988), a variant at codon 62 of the COMT gene. From now on, we will refer to the carriers of the Met allele as COMT MetCar compared to COMT Val/Val individuals. COMT Met/Met and COMT Val/Met were grouped due to the small sample size of each group. Regarding the three-marker Dys Hap (rs2619538-rs3213207-rs1047631) at the *DTNBP1* gene locus previously associated with a pattern of cognitive-related DLPFC functional activity consistent with reduced expression ^16, 17^, the LD-SNP rs9296989 replaced rs2619538 (D' = 0.96, R^2^ = 0.84). From now on, we will refer to the Dys Hap (T-A-G) carriers as Dys Hap+/- compared to Dys Hap +/+ individuals. Dys Hap +/− and Dys Hap −/− were grouped due to the small sample size of each group. For genotype frequencies, see Table S1.

## The N-back neuropsychological paradigm

The stimuli consist of numbers (range of 1-4) shown in random sequence and displayed in four corners of a diamond-shaped box. The task included a non-memory-guided condition (0-back), which presented the same stimuli but simply required subjects to identify the currently visible stimulus, and a working memory condition where subjects were instructed to recollect two (2-back) stimuli seen beforehand while continuing to encode additional incoming stimuli. The task alternated four 30-s blocks of a 0-back condition with four 30s blocks of the 2-back working memory condition. We assessed working memory performances before the fMRI session while individuals performed a computerized version of the N-back task outside the MRI scanner to limit the confounding effects of the scanning environment. We classified performance based on correct response during each working memory recollection block. We averaged the correct response count, reflecting the accuracy of working memory performance (Hit rate). Individuals with a Hit rate <25% were excluded ^6^. Reaction time has also been extracted (milliseconds). Individuals with reaction time <200ms were excluded ^18^. We compute an index of working memory efficiency as the ratio between accuracy and reaction time ^19^. Greater values of this index indicated better behavioral performance ^20^. Hit rate, reaction time, and efficiency rate differences were tested between sex groups through the Welch two-sample t test.

## MRI data acquisition and processing

MRI data were acquired using a standard quadrature head coil with a General Electric (Milwaukee, WI) 3.0 Tesla whole-body scanner. We used a whole-brain T1-weighted inversion recovery fast spoiled gradient recalled sequence (time repetition/time echo = 9.86/4.6ms; 124 contiguous axial slices; thickness = 1.3mm; voxel size = 0.9 × 0.9 × 1.3 mm; flip angle 6°; field of view 250 mm; matrix size 256 × 256; acquisition time = 6m 08s) for the individual brain structure acquisition. We used a gradient-echoplanar-imaging sequence (time repetition/time echo = 2000/30; 20 interleaved axial slices, thickness = 4mm, gap =1mm; voxel size 3.75 × 3.75 × 5mm; scans = 120; flip angle = 90°; field of view = 24cm; matrix = 64 × 64; acquisition time = 4m 12s) for the Blood-oxygen-level-dependent (BOLD) acquisition during the N-Back performance. MRI data underwent an individual quality check pipeline to retain only data unaffected by any technical artifact. Specifically, raw fMRI images and the raw T1 structural image were examined via visual inspection to detect artifacts like blurring, ringing, wrapping ^21, 22^, excessive noise, poor image contrast, and/or poor boundaries ^21, 23^. Regarding head coverage, T1 images were excluded in case of cropping. The crop of dorsal and frontal regions was also checked for fMRI images.

Individual raw T1 images were reoriented with the origin in the anterior commissure and the axial plane aligned along the anterior and posterior commissure axis (AC-PC line). Images were pre-processed with Statistical Parametric Mapping (SPM) version 12 (http://www.fil.ion.ucl.ac.uk/spm) and the Computational Anatomy Toolbox (CAT12, http://dbm.neuro.uni-jena.de/cat/). T1 segmentation served to estimate grey matter (GM), white matter (WM), and cerebrospinal fluid (CSF) ^24^. Bias correction removed intensity non-uniformities. Due to sex differences in brain size, customed brain reference templates for males and females have been suggested for MRI investigations focused on sex differences ^25-27^ to reduce the requirement for spatial deformation during image normalization and maintain a significant number of sex-related characteristics of individual brains ^28^. We built two customized templates for the individuals included in our MRI sample, one for the female sample and one for the male sample. Only individuals in the present study were included in the sex-specific template construction. We aimed to preserve the unique brain characteristics of each participant and closely align each individual's space with the corresponding sex template. We use the Multivariate Template Construction function implemented in the Advance Normalization toolbox – ANTs ^29^. We repeatedly run the algorithm for seven concatenated iterations ^30^. Each structural image was co-registered during the preliminary iteration (zero), and a temporary shared space template was computed. During the following iterations, all the individual structural images were (i) registered to the temporary shared-space template that had been generated one iteration before, (ii) then co-registered, and (iii) a new shared-space template was computed. We used a linear rigid registration through the Symmetric Normalization (SyN) transformation model ^29^ that maximizes the cross-correlation within the space of diffeomorphic maps across individuals.

The fMRI images were processed using SPM12. All functional images were individually examined and carefully screened for data quality using visual inspection for image artifacts, estimating indices for ghosting artifacts, and signal-to-noise ratio across the time series. Images were reoriented to the AC-PC line with the origin in the AC. Then, images were realigned to the mean image of the scan run, co-registered to the individual anatomical image, unwarped, and deformed to a 3 × 3 × 3mm^3^ voxel size. We performed a two-step normalization: (i) unwarped and deformed images were normalized into the sex-customed template space to minimize registration bias and maximize sensitivity to detect regional effects that can be impacted by registration errors; subsequentially, (ii) obtained images were normalized into the standard stereotactic Montreal Neurological Institute (MNI) space making them comparable across sex groups. We use the affine and non-linear transformation through the Symmetric Normalization function implemented in ANTs ^31^. Finally, images were smoothed using a 9-mm full-width at half-maximum isotropic 3D Gaussian kernel.

Concerning head motion correction, functional images were inspected to check for excessive motion correction (>3 mm in translation and >1.5 degrees in rotation). We also computed ensured that the movement of the head from one frame to the next during acquisition, measured through Framewise Displacement (FD), was lower than the published threshold (FD<0.05) ^32^. Additionally, twenty-four motion parameters were regressed to limit the effect of motion on connectivity estimates ^33^. fMRI responses were modeled using a canonical hemodynamic response function and temporally filtered using a 128 Hz high-pass filter to minimize scanner drift. Individual activity maps were created with t-statistics for the 2-back condition using the 0-back condition as a baseline using SPM12.

## Control fMRI analysis on sex-specific templates

Neuroimaging studies have shown structural differences between male and female brains ^34-36^. Generally, men have larger absolute volumes in different brain regions, ranging from 8% to 13%, with the highest differences in cerebrum and intracranial volume. Another study reported that the GM, WM, and CSF relative volume varies by sex, with men having a higher percentage of WM and CSF than women, while women have a higher rate of GM ^34^. Furthermore, males present higher variability than females ^37^. Such differences inevitably influence the normalization of individual images into standard templates, influencing group analysis on brain activity ^35^. The normalization methods aim to enable a meaningful comparison between individual brains utilizing standard templates, specifically the MNI template. This procedure necessitates adjusting each brain to conform to a consistent size, shape, and dimensions. This alignment is achieved by aligning each brain to the template, which involves warping, zooming, and shearing the images. Zooms can either reduce or enlarge the image dimensions, whereas shears involve stretching the diagonally opposite corners of the image away from each other. These transformations inevitably introduce various biases depending on how each brain differs from the template. To potentially mitigate biases stemming from the normalization, sex-specific templates have been considered ^38^. Such an approach could help preserve sex-specific characteristics while minimizing transformation-related biases. However, it is also plausible that sex-specific normalization procedures could introduce template-related effects into the analysis. To thoroughly examine any potential confounding effects originating from our two-step normalization procedure, we conducted additional control analyses:

- We analyzed the Dys Hap × COMT interaction independently for female and male groups on normalized fMRI images at the sex-specific template, i.e., step 1 of the normalization procedure described in section 5.
- We analyzed the Dys Hap × COMT interaction independently for female and male groups on normalized fMRI images at the MNI template, i.e., omitting step 1 of the normalization procedure described in section 5.
- We analyzed the sex × Dys Hap × COMT interaction independently on normalized fMRI images at the MNI template, i.e., omitting step 1 of the normalization procedure described in section 5.

Results of the control analyses showed (a) the Dys Hap × COMT significant interaction in both females (Brodmann Area 9; Z=3.31; k=24; p_uncorr_=0.0001) and males (Brodmann Area 9; Z=3.40; k=24; p_uncorr_=0.0001) analyzed independently on the sex-templates spaces. Weaker statistics are possibly related to the halved sample sizes. When analyzing Dys Hap × COMT interaction independently on females and males on normalized fMRI images at the MNI template, (b) results are preserved in females (Brodmann Area 9; k=13; MNI coord: x=-40 y=30 z=42; p_uncorr_=0.0001), but not in males, supporting our hypothesis for which using the standard MNI template might influence the sex-related effect because of transformations applied. Coherently, (c) we found no significant Sex × Dys Hap × COMT on normalized fMRI images at the MNI template. These findings indicate that omitting step 1 in the normalization procedure may introduce bias in the deformation of scans from native space to the standard MNI space by failing to account for differences in the distance of each individual's brain to the standard template, potentially eliminating sex-related characteristics ^39^.

2.7 Control fMRI analysis on age-related effects

Neuroimaging studies have frequently reported comparable brain activation of the prefrontal cortex during the N-back task in healthy adults both in terms of localization and intensity ^40, 41^, while age-related differences have been shown during neurodevelopment ^41, 42^ and ageing^42, 43^. Despite our sample comprising two subgroups of males and females within the same age range (males: 19 to 47 years old; females: 18 to 48 years old), we reported a significant difference between the two groups (two-sample t-test: t(206)=-2.07; p=0.04; Figure S4b). However, no significant main effect of age on DLPFC activity (F(201)=2.6, p_uncorr_=0.1) and no age by sex interaction (F(201)=0.03, p_uncorr_=0.9) have been reported by the voxel-wise analysis. However, to further disentangle the potential confounding effects of age in our sex groups, we performed a control analysis, repeating the three-way ANOVA in an age-matched subsample (N=204) where the age difference has been excluded (two-sample t-test: t(202)=-1.14; p=0.26; Figure S4c). The individuals assigned to the two sex groups were selected by the propensity score using the procedure implemented in the MatchIt R package (https://CRAN.R-project.org/package=MatchIt). The voxel-wise analysis performed on working memory related brain activity (categorical predictors: male vs female; COMT^Val/Val^ vs COMT^MetCar^; Dys Hap+/+ vs Dys Hap+/-; continuous predictors: age, IQ) confirms the absence of a significant main effect of age and age by sex interactions on DLPFC activity (p_TFCE-FEW_>0.05). On the other hand, a significant three-way interaction was preserved with the same localization of the effect found on the full sample (Brodmann Area 9; Z=3.41; k=34; *p_TFCE-FWE_=0.05; Figure S4a).

Also, the *post hoc* two-sample t-tests comparing DLPFC activity on all combinations of COMT genotypes and Dys Hap within the age-matched sex groups confirm our analysis on the full sample (Figure S4d). The results in females showed a reversed activation pattern as function of Dys Hap comparing COMT^Val/Val^ with higher DLPFC activity than COMT^MetCar^ in Dys+/- condition (t(23)=3.22;**p_FDR_=0.009), and COMTVal/Val with lower DLPFC activity than COMTMetCar in Dys+/+ condition (t(32)=3.63; **p_FDR_=0.005). On the other hand, results in males showed no significant differences comparing COMT^Val/Val^ and COMT^MetCar^ as a function of Dys Hap, however, DLPFC activity was lower in COMTVal/Val males with Dys Hap+/- compared with Dys Hap+/+ (t(23)=-3.20;**p_FDR_=0.009) in the opposite direction compared with the females’ activation pattern (t(27)=3.37;**p_FDR_=0.008). Therefore, these control analysis findings indicate that age differences within the two sex groups under consideration do not alter the impacts of Dys Hap and COMT concerning sex.

**Supplementary Figures**

Figure **S1. Ovariectomy validation. A.** Body weight monitored for 15 days. 3-Way ANOVA analysis revealed a main effect of Day F(7, 273)=4.349 ***p=0.0001, Surgery F(1,39)= 28.88***p<0.001 and genotype F(1,39)=20.50 **p<0.0001 as well as the interaction DayXGenotype F(7,273)=3.730 p=0.0007. No interaction DayXSurgery F(7,273)=1.631 p=0.1266, GenotypeXSurgery F(1, 39)= 0.9626 p=0.3326 or DayXSurgeryXGenotype F(7, 273)=1.369 p=0.2185 was detected. Multiple Comparisons was carried out with the uncorrected Fisher's LSD: ***p<0.001, **p<0.01 vs. Dys +/+ Sham, #p<0.05, ##p<0.01, ###p<0.001 vs. Dys +/- Sham. **B.** Uterus Weight/Body weight ratio. 2Way-ANOVA revealed a Surgery effect F(1,27)=65.16 ***p<0.0001, but not a Genotype effect F(1,27)=0.02435 p=0.8772 nor an interaction SurgeryXGenotype F(1,27)=0.00061 p=0.9804. Multiple Comparisons was carried out with the uncorrected Fisher's LSD ***p<0.001. All data are represented as mean ± S.e.m.

**Figure S2**. Representative full gel Western blot. A. COMT analyzed as total COMT (MB-COMT + S-COMT) quantified using densitometric analysis. B. GAPDH Western Blot used as housekeeping.


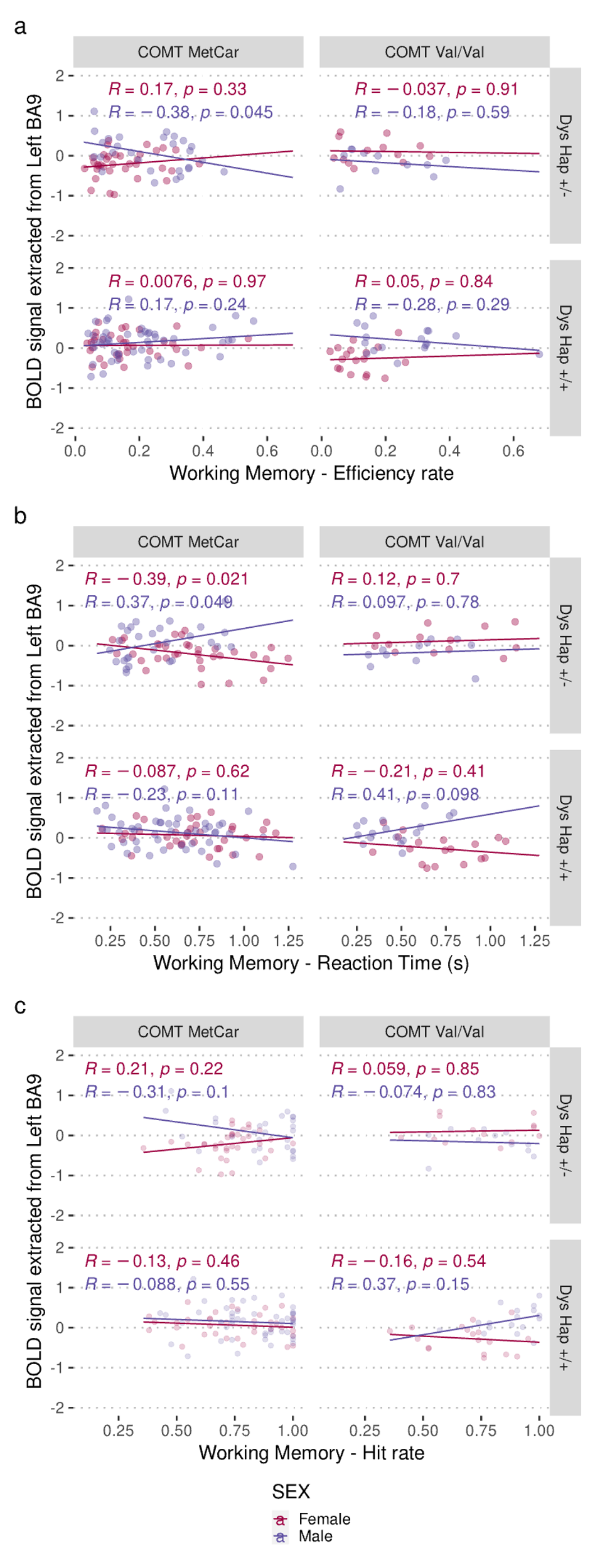


**Figure S3.** Scatterplots showing the association between BOLD estimates extracted from left BA9 and behavioral indices from the N-back task, i.e., the efficiency rate (A), the reaction time (in seconds, B), and the accuracy rate (C). All regression's r and p-values are reported in the figure.

**Figure S4.** Interaction between COMT rs4633, Dysbindin Hap, and sex on brain activity during the N-back task performance in the age-matched subsample. A. Brain multi-slice sections and rendering showing the significant DLPFC activity resulting from the three-way interaction between COMT genotype, Dys Hap, and sex during N-Back task located in the left DLPFC (Brodmann Area 9; MNI coordinates x=-30, y=32, z=41; k=32, Z=3.41; *pTFCE-FWE=0.05). The color bar indicates log_10_ TFCE scores ranging from 1.3 to 4. B. Density plot showing the age distribution in the full sample (N=211), where a significant age-difference has been reported between females and males through two sample t test. C. Density plot showing the age distribution in the age-matched subsample (N=204), where no significant age-differences have been reported between females and males through two sample t test. D. Box plots showing the differences between the COMT genotype and Dys Hap groups in the female and male groups assessed by the two-sample t test and corrected for multiple comparisons (k=6; pFDR<0.05). Only significant differences corrected for multiple comparisons have been marked with asterisks in the figure. Abbreviations: BOLD = Blood Oxygen Level-Dependent; BA9 = Brodmann Area 9.

**Figure S5.** Theoretical representation describing the impact of functional variants of DTNBP1 gene interacting with COMT on PFC-dependent cognitive functions across sexes in both mice and humans.

**Supplementary Tables**

**Table S1: Allele frequencies of COMT and Dys Hap.** Allele frequencies in the brain imaging study sample (Het), and at the population level (Population) and p value for the Hardy-Weinberg equilibrium (HWE) for each SNP in male and female groups.

|  | |  | Male | | | Female | | |
| --- | --- | --- | --- | --- | --- | --- | --- | --- |
| Marker | Allele | | Freq. (Het) | Freq. (Population) | HWE p value | Freq. (Het) | Freq. (Population) | HWE p value |
| rs4633 | T | C | 0.47 | 0.50 | 0.57 | 0.42 | 0.50 | 0.12 |
| s1047631 | C | T | 0.20 | 0.20 | 1 | 0.28 | 0.27 | 1 |
| rs3213207 | C | T | 0.12 | 0.13 | 0.41 | 0.22 | 0.21 | 1 |
| rs9296989 | G | A | 0.52 | 0.69 | 1 | 0.44 | 0.49 | 0.31 |

**References**

1. Cora MC, Kooistra L, Travlos G. Vaginal cytology of the laboratory rat and mouse: review and criteria for the staging of the estrous cycle using stained vaginal smears. *Toxicologic pathology* 2015; **43**(6)**:** 776-793.

2. Collado-Torres L, Burke EE, Peterson A, Shin J, Straub RE, Rajpurohit A *et al.* Regional Heterogeneity in Gene Expression, Regulation, and Coherence in the Frontal Cortex and Hippocampus across Development and Schizophrenia. *Neuron* 2019; **103**(2)**:** 203-216 e208.

3. Pergola G, Parihar M, Sportelli L, Bharadwaj R, Borcuk C, Radulescu E *et al.* Consensus molecular environment of schizophrenia risk genes in coexpression networks shifting across age and brain regions. *Sci Adv* 2023; **9**(15)**:** eade2812.

4. Pergola G, Di Carlo P, Jaffe AE, Papalino M, Chen Q, Hyde TM *et al.* Prefrontal Coexpression of Schizophrenia Risk Genes Is Associated With Treatment Response in Patients. *Biol Psychiatry* 2019; **86**(1)**:** 45-55.

5. Antonucci LA, Di Carlo P, Passiatore R, Papalino M, Monda A, Amoroso N *et al.* Thalamic connectivity measured with fMRI is associated with a polygenic index predicting thalamo-prefrontal gene co-expression. *Brain Struct Funct* 2019; **224**(3)**:** 1331-1344.

6. Fazio L, Pergola G, Papalino M, Di Carlo P, Monda A, Gelao B *et al.* Transcriptomic context of DRD1 is associated with prefrontal activity and behavior during working memory. *Proc Natl Acad Sci U S A* 2018; **115**(21)**:** 5582-5587.

7. Pergola G, Di Carlo P, D'Ambrosio E, Gelao B, Fazio L, Papalino M *et al.* DRD2 co-expression network and a related polygenic index predict imaging, behavioral and clinical phenotypes linked to schizophrenia. *Translational psychiatry* 2017; **7**(1)**:** e1006-e1006.

8. Taurisano P, Pergola G, Monda A, Antonucci LA, Di Carlo P, Piarulli F *et al.* The interaction between cannabis use and a CB1-related polygenic co-expression index modulates dorsolateral prefrontal activity during working memory processing. *Brain Imaging Behav* 2021; **15**(1)**:** 288-299.

9. Kumari S, Nie J, Chen HS, Ma H, Stewart R, Li X *et al.* Evaluation of gene association methods for coexpression network construction and biological knowledge discovery. *PLoS One* 2012; **7**(11)**:** e50411.

10. Jaffe AE, Straub RE, Shin JH, Tao R, Gao Y, Collado-Torres L *et al.* Developmental and genetic regulation of the human cortex transcriptome illuminate schizophrenia pathogenesis. *Nat Neurosci* 2018; **21**(8)**:** 1117-1125.

11. Taliun D, Harris DN, Kessler MD, Carlson J, Szpiech ZA, Torres R *et al.* Sequencing of 53,831 diverse genomes from the NHLBI TOPMed Program. *Nature* 2021; **590**(7845)**:** 290-299.

12. Fuchsberger C, Abecasis GR, Hinds DA. minimac2: faster genotype imputation. *Bioinformatics* 2015; **31**(5)**:** 782-784.

13. Purcell S, Neale B, Todd-Brown K, Thomas L, Ferreira MA, Bender D *et al.* PLINK: a tool set for whole-genome association and population-based linkage analyses. *Am J Hum Genet* 2007; **81**(3)**:** 559-575.

14. Howie B, Marchini J, Stephens M. Genotype imputation with thousands of genomes. *G3 (Bethesda)* 2011; **1**(6)**:** 457-470.

15. Delaneau O, Marchini J, Zagury JF. A linear complexity phasing method for thousands of genomes. *Nat Methods* 2011; **9**(2)**:** 179-181.

16. Papaleo F, Burdick MC, Callicott JH, Weinberger DR. Epistatic interaction between COMT and DTNBP1 modulates prefrontal function in mice and in humans. *Mol Psychiatry* 2014; **19**(3)**:** 311-316.

17. Scheggia D, Mastrogiacomo R, Mereu M, Sannino S, Straub RE, Armando M *et al.* Variations in Dysbindin-1 are associated with cognitive response to antipsychotic drug treatment. *Nat Commun* 2018; **9**(1)**:** 2265.

18. Bertolino A, Caforio G, Petruzzella V, Latorre V, Rubino V, Dimalta S *et al.* Prefrontal dysfunction in schizophrenia controlling for COMT Val158Met genotype and working memory performance. *Psychiatry research* 2006; **147**(2-3)**:** 221-226.

19. Pergola G, Di Carlo P, Andriola I, Gelao B, Torretta S, Attrotto MT *et al.* Combined effect of genetic variants in the GluN2B coding gene (GRIN2B) on prefrontal function during working memory performance. *Psychol Med* 2016; **46**(6)**:** 1135-1150.

20. Taurisano P, Antonucci LA, Fazio L, Rampino A, Romano R, Porcelli A *et al.* Prefrontal activity during working memory is modulated by the interaction of variation in CB1 and COX2 coding genes and correlates with frequency of cannabis use. *Cortex* 2016; **81:** 231-238.

21. Lu W, Dong K, Cui D, Jiao Q, Qiu J. Quality assurance of human functional magnetic resonance imaging: a literature review. *Quant Imaging Med Surg* 2019; **9**(6)**:** 1147-1162.

22. Wood ML, Henkelman RM. Truncation artifacts in magnetic resonance imaging. *Magn Reson Med* 1985; **2**(6)**:** 517-526.

23. Song Z, Tustison N, Avants B, Gee JC. Integrated graph cuts for brain MRI segmentation. *Med Image Comput Comput Assist Interv* 2006; **9**(Pt 2)**:** 831-838.

24. Ashburner J, Friston KJ. Unified segmentation. *Neuroimage* 2005; **26**(3)**:** 839-851.

25. De Bellis MD, Keshavan MS, Beers SR, Hall J, Frustaci K, Masalehdan A *et al.* Sex differences in brain maturation during childhood and adolescence. *Cereb Cortex* 2001; **11**(6)**:** 552-557.

26. Evans TM, Flowers DL, Napoliello EM, Eden GF. Sex-specific gray matter volume differences in females with developmental dyslexia. *Brain Struct Funct* 2014; **219**(3)**:** 1041-1054.

27. Gennatas ED, Avants BB, Wolf DH, Satterthwaite TD, Ruparel K, Ciric R *et al.* Age-Related Effects and Sex Differences in Gray Matter Density, Volume, Mass, and Cortical Thickness from Childhood to Young Adulthood. *J Neurosci* 2017; **37**(20)**:** 5065-5073.

28. Fonov V, Evans AC, Botteron K, Almli CR, McKinstry RC, Collins DL *et al.* Unbiased average age-appropriate atlases for pediatric studies. *Neuroimage* 2011; **54**(1)**:** 313-327.

29. Avants BB, Epstein CL, Grossman M, Gee JC. Symmetric diffeomorphic image registration with cross-correlation: evaluating automated labeling of elderly and neurodegenerative brain. *Med Image Anal* 2008; **12**(1)**:** 26-41.

30. Avants BB, Duda JT, Kilroy E, Krasileva K, Jann K, Kandel BT *et al.* The pediatric template of brain perfusion. *Sci Data* 2015; **2:** 150003.

31. Avants BB, Tustison N, Song G. Advanced normalization tools (ANTS). *Insight j* 2009; **2:** 1-35.

32. Power JD, Barnes KA, Snyder AZ, Schlaggar BL, Petersen SE. Spurious but systematic correlations in functional connectivity MRI networks arise from subject motion. *Neuroimage* 2012; **59**(3)**:** 2142-2154.

33. Friston K. Learning and inference in the brain. *Neural Netw* 2003; **16**(9)**:** 1325-1352.

34. Ritchie SJ, Cox SR, Shen X, Lombardo MV, Reus LM, Alloza C *et al.* Sex Differences in the Adult Human Brain: Evidence from 5216 UK Biobank Participants. *Cereb Cortex* 2018; **28**(8)**:** 2959-2975.

35. Cosgrove KP, Mazure CM, Staley JK. Evolving knowledge of sex differences in brain structure, function, and chemistry. *Biol Psychiatry* 2007; **62**(8)**:** 847-855.

36. Ruigrok AN, Salimi-Khorshidi G, Lai MC, Baron-Cohen S, Lombardo MV, Tait RJ *et al.* A meta-analysis of sex differences in human brain structure. *Neurosci Biobehav Rev* 2014; **39**(100)**:** 34-50.

37. Wierenga LM, Doucet GE, Dima D, Agartz I, Aghajani M, Akudjedu TN *et al.* Greater male than female variability in regional brain structure across the lifespan. *Hum Brain Mapp* 2022; **43**(1)**:** 470-499.

38. Ramzanpour M, Jafari B, Smith J, Allen J, Hajiaghamemar M. Comprehensive study of sex-based anatomical variations of human brain and development of sex-specific brain templates. *Brain Multiphysics* 2023; **4**.

39. Yang G, Zhou S, Bozek J, Dong HM, Han M, Zuo XN *et al.* Sample sizes and population differences in brain template construction. *Neuroimage* 2020; **206:** 116318.

40. Rottschy C, Langner R, Dogan I, Reetz K, Laird AR, Schulz JB *et al.* Modelling neural correlates of working memory: a coordinate-based meta-analysis. *Neuroimage* 2012; **60**(1)**:** 830-846.

41. Owen AM, McMillan KM, Laird AR, Bullmore E. N-back working memory paradigm: a meta-analysis of normative functional neuroimaging studies. *Hum Brain Mapp* 2005; **25**(1)**:** 46-59.

42. Yaple Z, Arsalidou M. N-back Working Memory Task: Meta-analysis of Normative fMRI Studies With Children. *Child Dev* 2018; **89**(6)**:** 2010-2022.

43. Turner GR, Spreng RN. Executive functions and neurocognitive aging: dissociable patterns of brain activity. *Neurobiol Aging* 2012; **33**(4)**:** 826 e821-813.
